# Development of a fluorescence-based method for the rapid determination of Zika virus polymerase activity and the screening of antiviral drugs

**DOI:** 10.1038/s41598-019-41998-1

**Published:** 2019-04-01

**Authors:** Yanira Sáez-Álvarez, Armando Arias, Carmen del Águila, Rubén Agudo

**Affiliations:** 1 Facultad de Farmacia, Universidad San Pablo-CEU, CEU Universities, Madrid, 28668 Spain; 2 Life Science & Bioengineering Building, Technical University of Denmark, 2800 Kongens Lyngby, Denmark

## Abstract

Zika virus (ZIKV) is an emerging pathogen that has been associated with large numbers of cases of severe neurologic disease, including Guillain-Barré syndrome and microcephaly. Despite its recent establishment as a serious global public health concern there are no licensed therapeutics to control this virus. Accordingly, there is an urgent need to develop methods for the high-throughput screening of antiviral agents. We describe here a fluorescence-based method to monitor the real-time polymerization activity of Zika virus RNA-dependent RNA polymerase (RdRp). By using homopolymeric RNA template molecules, *de novo* RNA synthesis can be detected with a fluorescent dye, which permits the specific quantification and kinetics of double-strand RNA formation. ZIKV RdRp activity detected using this fluorescence-based assay positively correlated with traditional assays measuring the incorporation of radiolabeled nucleotides. We also validated this method as a suitable assay for the identification of ZIKV inhibitors targeting the viral polymerase using known broad-spectrum inhibitors. The assay was also successfully adapted to detect RNA polymerization activity by different RdRps, illustrated here using purified RdRps from hepatitis C virus and foot-and-mouth disease virus. The potential of fluorescence-based approaches for the enzymatic characterization of viral polymerases, as well as for high-throughput screening of antiviral drugs, are discussed.

## Introduction

Zika virus (ZIKV) is an emerging human pathogen of the *Flaviviridae* family, a group of single-stranded (ss) RNA enveloped viruses. Members of this family also include the human pathogens dengue virus, yellow fever virus, West Nile virus, tick-borne encephalitis virus, Japanese encephalitis virus and hepatitis C virus (HCV)^1^. ZIKV is an arthropod-borne virus and transmission is primarily caused by the bite of infected *Aedes* species mosquitoes^2^, but it can also be spread perinatally^3^, sexually^4^ or by blood transfusions^5^. ZIKV infection in humans is generally asymptomatic^6,7^; however, a significant proportion of infected individuals (~20%) develop neurological conditions, including Guillain-Barré syndrome (GBS), which is the most frequent cause of acute flaccid paralysis not associated with poliovirus in adults, and microcephaly, in newborns. An increase in the incidence of GBS and microcephaly has been associated with outbreaks of ZIKV in Micronesia (2007), French Polynesia (2013), and Brazil (2015)^8–10^. Accordingly, the World Health Organization Public Health Emergency Committee declared ZIKV a global public health emergency of international concern^11^.

ZIKV has a positive-sense ssRNA genome of approximately 10.8 kb in length, which encodes a single polyprotein of 3400 amino acids flanked by untranslated RNA regions^12–14^. The polyprotein undergoes successive proteolytic processing to generate three structural proteins: the capsid protein, the precursor of the membrane protein and the envelope protein, as well as seven nonstructural proteins (NS1, NS2A, NS2B, NS3, NS4A, NS4B and NS5). The different non-structural proteins are involved in the essential steps of the viral replication cycle inside the host cell. Among them, NS5 is the largest (903 amino acids) and most conserved viral protein^15^. NS5 includes an N-terminal domain containing methyltransferase activity (residues 1–262) and a C-terminal RNA-dependent RNA polymerase (RdRp) domain (residues 275–903). A short linker interdomain formed by residues 263–274 covalently connects both enzymatic activities^16,17^. The crystal structures of the whole ZIKV NS5 protein and the RdRp domain alone have been recently solved^16–18^. ZIKV RdRp exhibits a typical encircled right-hand configuration with palm, fingers and thumb subdomains, and six conserved motifs (A–F) that are common to other viral RdRps. These motifs are critical for its polymerase activity, as they are involved in RNA and nucleotide binding, coordination of metal ions, and catalysis^19^. The catalytic aspartates are located in conserved motifs A (D536) and C (GDD tract at positions 665–667). These aspartates constitute the catalytic triad responsible for nucleotide transfer to nascent RNA. The process entails the coordination of two divalent cations by these residues that are essential to the catalytic process^20^.

Owing to substantial differences in the mechanisms of replication in RNA viruses and the host cell – RNA-templated RNA synthesis *versus* conventional DNA-dependent DNA synthesis – viral RdRps are key targets for direct-acting antiviral agents^21^. The recent development of nucleoside and non-nucleoside analogs (NAI and NNI, respectively) targeting RdRps of diverse members of the *Flaviviridae* family have generated encouraging results^22–29^, including sofosbuvir, the first HCV RdRp NAI approved by the U.S. Food and Drug Administration for its potent antiviral efficacy and good tolerance in humans^30^. While NNIs typically require no intracellular modification to elicit their inhibitory activity, as they bind directly to allosteric sites on RdRps, NAIs generally require phosphorylation by the host cell machinery to be active. Phosphorylated nucleoside analogs are thus able to bind to the RdRp active site and compete with natural NTPs for their incorporation into the nascent RNA. Despite recent significant progress, no drugs are yet licensed for the treatment of disease caused by any mosquito-borne *flavivirus*, including ZIKV (for a comprehensive analysis of this issue see^31–33^).

In this study, we outline the development of a reliable method for rapid detection of ZIKV polymerase activity in real-time. We show that recombinant ZIKV RdRp can synthesize RNA *de novo*, which can be then quantified in real-time using a fluorescent dye (SYTO 9), which detects the presence of double-stranded (ds) RNA generated after the copy of single-stranded homopolymeric RNA. We also demonstrate the potential value of this protocol to screen antiviral compounds by using known broad-spectrum antivirals, which reproduce their inhibitory activities in this assay. The versatility of this method was further established with RdRps from two distinct viruses: foot-and-mouth disease virus (FMDV) and hepatitis C virus (HCV). The implications of these results for high-throughput approaches are discussed.

## Methods

### Reagents

Electro-competent *Eschericia coli* BL21(DE3)-pRIL cells were produced according to standard protocols^34^. The following reagents were purchased from Applichem: LB medium (powder), ampicillin (used at 100 μg/mL) and chloramphenicol (used at 17 μg/mL), MnCl_2_, ammonium acetate, NaCl, MgCl_2_, ZnCl_2_, glycerol, imidazole, isopropyl-β-D-1-thiogalactopyranoside (IPTG), 1,4-dithiothreitol (DTT), phenylmethanesulfonyl fluoride (PMSF) and Tris base. *Dpn*I restriction enzyme, BSA, Phusion^®^ High-Fidelity (HF) DNA polymerase and Gibson Assembly^®^ Master Mix were obtained from New England Biolabs. Stock solutions of ATP and GTP, SYTO^TM^ 9 green fluorescent dye and HisPur^TM^ Ni-NTA resin were purchased from ThermoFisher Scientific. pET28a and pET16b expression vectors were obtained from Novagen. Nucleotide analogs ribavirin 5′-triphosphate and cordycepin 5′-triphosphate were obtained from Jena Bioscience and Sigma-Aldrich, respectively. SYBR^®^ Green II fluorescent dye, Tween 20, heparin, EDTA and oligonucleotides were also purchased from Sigma-Aldrich. ssRNA polyuridylic acid (poly-U), ssRNA polyadenylic acid (poly-A) and ssRNA polycytidylic acid (poly-C) were purchased from Amersham Biosciences. Radiolabeled nucleotides [α-^32^P]ATP and [α-^32^P]GTP (250 μCi; 3000 Ci/mmol) were obtained from Perkin Elmer. FMDV viral protein genome-linked 1 (VPg1) was synthetically prepared as previously described^35^.

### Construction of expression vectors

The ZIKV NS5 RdRp domain (spanning amino acid residues 275–903^16^) from isolate PRVABC59 (Puerto Rico 2015, ATCC ref. number VR-1843)^13^ was cloned into the pET16b expression plasmid between *Nde*I and *Xho*I sites using the Gibson assembly method. To do this, the vector backbone was amplified using pET16b as template; the PCR reaction contained 1 × Phusion HF buffer, 200 μM dNTPs, 0.5 units of Phusion^®^ HF DNA polymerase and 0.25 μM of a specific pET16_Fw and pET16_rv primer pair (Supplementary Table S1). The amplification conditions used were as follows: 98 °C (3 min), 30 cycles of 98 °C (15 s), 51 °C (20 s) and 72 °C (4 min) each, and 10 min of elongation at 72 °C. The RdRp domain was amplified from a pcDNA vector containing the full-length *NS5* gene. PCR amplification was similar to that described above but using the specific primers NS5 short_pET16_Fw and NS5_pET16_rv (Supplementary Table S1). After purification, the vector and insert were mixed in the presence of 2 × Gibson Assembly Master Mix and the assembly reaction was carried out following the recommendations of the manufacturer. The assembled product was transformed into *E. coli* BL21(DE3)-pRIL cells. After plasmid extraction from three independent bacterial colonies, nucleotide sequencing determined that two DNA samples contained the correct construct. The resulting plasmid pET16a-ZIKV-NS5RdRp encodes for ZIKV RdRp fused to an HHHHHHHHHHSSGHIEG amino acid tract in its N-terminus that is used for affinity purification using HisPur^TM^ Ni-NTA resin The predicted molecular weight of this protein is 75 kDa.

A catalytically inactive enzyme was prepared by site-directed mutagenesis of the pET16a-ZIKV-NS5RdRp plasmid, encoding for substitutions D665N and D666N in the active site, which affects two catalytic Asp residues. The amplification reagents were the same as above with primers NS5_GNN_Fw and NS5_GNN_rv (Supplementary Table S1) and pET16a-ZIKV-NS5RdRp as template. PCR reaction conditions used were 98 °C (3 min), 30 cycles of 98 °C (15 s), 54 °C (20 s) and 72 °C (4 min), followed by an elongation step of 10 min at 72 °C. The resulting expression plasmid was termed pET16a-ZIKV-NS5RdRp-GNN.

Plasmids pET-28a3Dpol and pET-28aD338A, which encode wild-type and catalytically inactive (D338A) FMDV 3D polymerases, were obtained in previous studies^35,36^.

The plasmid for the expression of HCV NS5B polymerase was prepared using the Gibson assembly method as described above. Briefly, the pET28a vector backbone was amplified by PCR using primers pET28a_Fw and pET28a_rv (Supplementary Table S1). An insert containing the HCV polymerase sequence was obtained by PCR amplification of plasmid Jc1FLAG2(p7-nsGluc2A)^37^, using primers NS5B_HCV_Fw and NS5B_Δ21_HCV_rv (Supplementary Table S1). Both vector and insert were assembled as described above. The resulting plasmid, termed pET28-HCV NS5bΔ21, encodes for HCV NS5b polymerase lacking the most C-terminal 21 amino acids and containing a C-terminal His-tag (LEHHHHHH). The predicted molecular weight of this recombinant protein is 65 kDa. For the construction of pET28-HCV NS5bΔ21-GNN (expressing a catalytically-inactive RdRp) we used Gibson assembly and plasmid Jc1FLAG2(p7-nsGluc2a)/GNN^37^ to generate the insert. All the constructs were analyzed by sequencing to confirm the presence of the expected insert and the absence of undesired mutations.

### Expression and purification of viral polymerases

For the expression of ZIKV NS5 RdRp (hereafter referred to as ZIKV RdRp), *E. coli* cells were transformed by electroporation with pET16a-ZIKV-NS5RdRp. Single kanamycin and chloramphenicol resistant colonies were cultured overnight in 10 mL of LB in the presence of antibiotics at 37 °C. Each culture was then inoculated into 200 mL of LB with antibiotics and incubated at 37 °C. When an optical density at 600 nm of 0.7 was reached, 500 μM IPTG, 50 μM MgCl_2_ and 50 μM ZnCl_2_ were added to the culture, which was incubated at 30 °C for 4 additional hours. Cells were then pelleted by centrifugation at 5,000 rpm for 15 min at 4 °C and stored at −80 °C until further use.

The bacterial pellets recovered from 200 mL cultures were resuspended in 20 mL of lysis buffer [50 mM Tris HCl, pH 8.0, 300 mM NaCl, 400 mM ammonium acetate, 4 mM MgCl_2_, 10%, glycerol, 10 mM imidazole, and 0.1% (v/v) Tween 20] and sonicated on ice for 6 cycles of 20 s alternating with 5 cycles of 10 s. Cell debris was pelleted at 11,000 rpm for 30 min at 4 °C and the supernatant mixed with 800 μL of Ni-NTA resin previously equilibrated with 20 volumes of lysis buffer without ammonium acetate (BWE buffer). The lysate was incubated with the resin in batch method with gentle mixing during 1 h at 4 °C. The unbound fraction was then removed by decantation and the resin was then loaded onto a column and extensively washed with 20 × column volumes of BWE buffer and 20 × column volumes of BWE buffer containing 25 mM imidazole. The resin was further washed with increasing concentrations of imidazole (successive one-column volumes of BWE buffer containing 50, 60, 70, 80, 90, 100 and 125 mM imidazole). Finally, the His-tagged protein was eluted in 400 μL of BWE buffer containing 400 mM imidazole. The sample was dialyzed for 3 hours at 4 °C against 200 volumes of dialysis buffer [50 mM Tris-HCl, pH 8.0, 150 mM NaCl, 5 mM MgCl_2_, 10%, glycerol, 1 mM DTT and 0.05% (v/v) Tween 20]. Samples obtained from different purification batches were pooled, quantified, aliquoted and stored at −80 °C until further use. Expression and purification of the recombinant ZIKV NS5RdRp-GNN (with D665N and D666N substitutions) was carried out following the same protocol. Likewise, the expression and purification of HCV NS5bΔ21 and NS5bΔ21-GNN polymerases was carried out following the same protocol described for ZIKV NS5. The protocol for the expression and purification of FMDV 3D polymerases has been described previously^35,36^.

### Fluorescence-based activity assay for ZIKV RdRp

For the detection of RNA synthesis by ZIKV RdRp, we established a real-time assay based on the fluorescent dye SYTO 9, which binds dsRNA but not ssRNA template molecules. The fluorescence emitted was recorded in real-time using a Fluostar Optima fluorimeter (BMG Labtech) using excitation and emission filters at 485 and 520 nm, respectively. The assay records the synthesis of dsRNA in a reaction using a poly-U molecule as a template and ATP as the nucleotide substrate. This technique has been adapted from methods previously documented for the detection of DNA synthesis^38^.

Reactions were performed in individual wells of black 96-well flat-bottom plates. The standard reaction contained 50 mM Tris-HCl, pH 7.5, 2.5 mM MnCl_2_, 500 μM ATP, 20 μg/mL poly-U, 0.1 mg/mL BSA and 0.25 μM SYTO 9 (50 μM stock solution in TE buffer pH 7.5). The assay was initiated by the addition of 250 nM ZIKV RdRp and the fluorescence was recorded over 30 min at 30 °C.

Variations on this assay, for example, different concentrations of reagents and/or the presence of additional compounds, are specifically indicated in each corresponding section. For graphical representation, background fluorescence obtained at time point 0 was subtracted from each value.

To determine K_m_ and V_max_ constants for ZIKV RdRp binding to poly-U ssRNA, standard reactions were carried out in increasing concentrations of the template (0.5–50 μg/mL) in the presence of ATP at 500 μM. The kinetic parameters for ATP were obtained from assays in the presence of increasing concentrations of this nucleotide (200–2250 μM) and using 3 μg/mL of poly-U.

IC_50_ values were obtained from standard reactions carried out in the presence of 3 μg/mL poly-U and 1500 μM ATP, and increasing concentrations of each inhibitor.

End-point fluorometric reactions were performed in black 96-well black-flat bottom plates at 30 °C in the presence of the same reagents as described above, but in the absence of dye. The reactions were quenched at 60 min by adding in 25 mM EDTA to the samples. Either SYTO9 or SYBR Green II dye was then added to the sample (0.25 μM or 1x, respectively) and the mix reaction was incubated at room temperature for 5 min to allow the stabilization of RNA-dye complexes and fluorescence emission. To determine background fluorescence levels, a negative control was assayed in parallel, where the reaction was quenched before adding ZIKV RdRp. The quenched control reaction was incubated for 1 h at 30 °C, and then 0.25 μM SYTO9 or 1 × SYBR Green II, respectively, was added to the sample and fluorescence recorded as described above.

### Real-time fluorescence-based assay for HCV NS5bΔ21 and FMDV 3D polymerases

Polymerase activity assays with HCV NS5bΔ21 were performed as described above for ZIKV RdRp. For the detection of FMDV 3D polymerase activity, 49 μL of reaction mix [50 mM Tris-HCl, pH 7.5, 0.6 mM MnCl_2_, 33 mM NaCl, 100 μM UTP, 40 μg/mL poly-A, 8% glycerol (v/v), 150 μM VPg1, 0.1 mg/mL BSA and 0.25 μM of SYTO 9 (50 μM stock solution in TE buffer, pH 7.5)] was added to each well of a black 96-well flat-bottom plate. Reactions were initiated by the addition of 1 μL of FMDV 3D (1 μM final concentration) and plates were incubated at 37 °C for 30 min. Fluorescence intensity was recorded as above.

### ZIKV NS5 RdRp domain and HCV NS5bΔ21 polymerization assays using radiolabeled nucleotides

Similar procedures were used for both ZIKV NS5 RdRp domain and HCV NS5bΔ21 polymerases. Radioactivity assays were performed in 10 μL reactions containing 50 mM Tris-HCl, pH 7.5, 2.5 mM MnCl_2_, 0.1 mg/mL BSA, and either 500 μM ATP, 16 nM [α-^32^P]ATP and 40 μg/mL poly-U; or 500 μM GTP, 16 nM [α-^32^P]GTP and 20 μg/mL poly-C. Reactions were performed at 30 °C and initiated by addition of of 1 μL of either ZIKV or HCV recombinant polymerases to reach a final concentration of 1 μM in the assay. The reaction was stopped at different time points by addition of 2 μL of formamide loading buffer [10 mM EDTA, 95% (v/v) formamide, 0.03% (w/v), xylene-cyanol, final concentration]. The reaction products were resolved by electrophoresis in 20% polyacrylamide gels containing 1 × TBE buffer and 8 M urea, at 30 W for 120 min. *De novo* synthesized polynucleotides using poly-U and [α-^32^P]ATP as substrates were detected either by autoradiography or by phosphorimaging (Phosphorimager BAS 1500, Fuji).

### Data analysis

Fluorometric results were expressed as mean ± SD. Statistical significance was analyzed by two-way ANOVA using GraphPad Prism, version 7, as specified in the figure legends. K_m_ determinations were obtained by plotting the velocity of the reaction as a function of nucleotide or ssRNA template concentrations using nonlinear regression. IC_50_ values were obtained by fitting the velocity data to a four-parameter logistic equation. Kinetic parameters and IC_50_ values were calculated using Sigmaplot, version 11. Z’ factor was calculated according to Zhang *et al*.^39^: Z’ = 1 − [(3SD_c+_ + 3SD_c−_)/(mean_c+_ − mean_c−_)] where “c+” is the activity obtained in a standard assay and “c−” is the nonspecific activity obtained in a control performed in the absence of MnCl_2_.

## Results

### Purification and biochemical characterization of recombinant ZIKV RdRp

**Figure 1:**
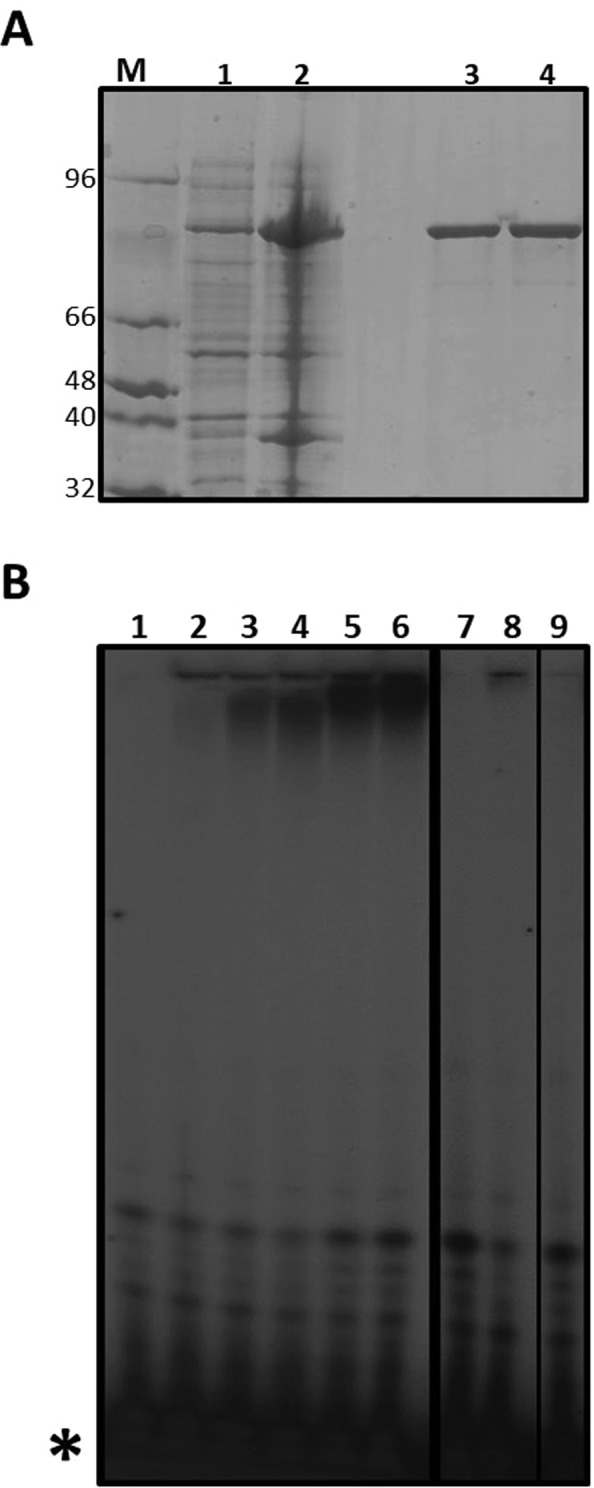
Purification of ZIKV NS5 RdRp and determination of polymerase activity. (**A**) SDS-PAGE analysis of ZIKV NS5 RdRp domain expressed in *E. coli* and purified by affinity chromatography. Lysates of *E. coli* BL21(DE3)-pRIL containing plasmid pET16a-ZIKV-NS5 and grown in the absence (lane 1) or presence (lane 2) of 500 μM IPTG. ZIKV RdRp wild-type (lane 3) and ZIKV RdRp GNN inactive mutant (lane 4) were obtained after purification through Ni-NTA resin. M, molecular marker. The molecular weight of each band is indicated (in kDa). Procedures for protein expression and purification are described in Methods. (**B**) Representative electropherogram of polymerization reactions carried out by ZIKV RdRp in the presence of [α-^32^P]ATP, using poly-U as template and MnCl_2_ as a metal donor. Reactions were stopped at increasing time points (from 0 to 3 hours; lanes 2 to 6). Polymerization assays performed in the absence of enzyme (lane 1), in the presence of MgCl_2_ as metal donor (lane 7), in the absence of any metal donor (lane 8), or using the GNN polymerase mutant (lane 9) are shown. Gel electrophoresis and visualization by autoradiography analysis were performed as described in Methods. The position of labeled nucleotides not incorporated in the RNA is indicated (*). A thick vertical line separates images obtained from independent gels. Both gels were run and autoradiographied using the same experimental conditions. A thinner black line separates two images cropped from the same gel but which were located in non-contiguous positions.

The RdRp domain of ZIKV NS5 and a catalytic inactive mutant (GNN) were purified as described in Methods. Recombinant proteins were ≥95% pure as judged by PAGE analysis and Coomassie brillant blue R-250 staining (Fig. 1A).

The overexpression and biochemical characterization of ZIKV RdRp under different experimental conditions has been previously published^40–42^. For the preliminary evaluation of ZIKV NS5 RdRp domain activity *in vitro*, we adapted a polymerization assay based on the detection of radioactive nucleotides incorporated by the polymerase. This method makes use of a homopolymeric ssRNA as a template in the absence of any primer, since it has been previously demonstrated that ZIKV NS5 can initiate RNA synthesis *de novo*^42^. The reactions were performed in the presence of radioactive-labeled nucleotides, and polymerization products were resolved by PAGE. RNA synthesis in the absence of primer was observed both in the presence of poly-U and [α-^32^P]ATP as template and nucleotide substrates (Fig. 1B), and in the presence of poly-C and [α-^32^P]GTP (Fig. S1). We observed polymerization activity *de novo* in the presence of Mn^2+^ but not Mg^2+^, in agreement with a previous observation^41^ (Fig. 1B). The same reaction in the presence of the catalytically-inactive mutant GNN showed no detectable signal (Fig. 1B, lanes 7 to 9).

Previous studies suggested that, under certain circumstances, *flaviviral* polymerases can catalyze the terminal transference of nucleotides to RNA. However, this transferase activity has never been reported for ZIKV RdRp^42^. To rule out the possibility that the incorporation of nucleotides detected in our assay was due to the terminal transference of nucleotides and not to *de novo* RNA synthesis (as we expect), we performed the same assay but in the presence of radioactive nucleotides that were less competent for viral RNA synthesis: [α-^32^P]GTP to poly-U and [α-^32^P]ATP to poly-C. As shown in Fig. S2B, no elongation was detected under these conditions, supporting the notion that the activity detected was due to *de novo* RNA synthesis. Thus, these results show that both homopolymeric templates, poly-C and poly-U, can be used by ZIKV to initiate RNA replication, as has been previously documented^42^.

### Detection of ZIKV RdRp polymerization activity by fluorometric assays in real time

Based on the above results, we next sought to detect polymerization activity using a fluorescence-detection method. For this aim, we attemped to establish an assay to quantify RNA synthesis activity as the relative increase in fluorescence emitted by SYBR Green II dye after binding to dsRNA. This procedure was adapted from methods previously described to detect dsDNA synthesis by the human primase-polymerase PrimPol^38^. We anticipated that binding of this intercalating agent to dsRNA generated by ZIKV RdRp polymerization activity would lead to an increase in the emitted fluorescence.

**Figure 2:**
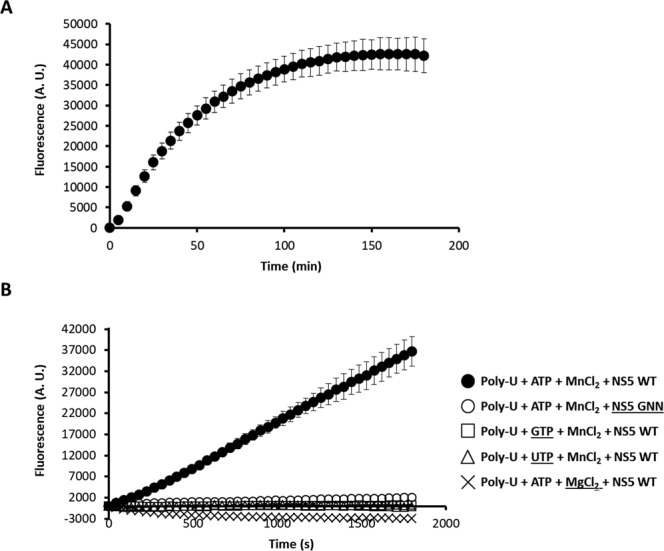
Fluorescence-based polymerization assay using ZIKV NS5 RdRp. (**A**) Polymerization activity was recorded as the relative increase in fluorescence over 180 min in the presence of ZIKV NS5. Shown are the average values of three independent experiments. (**B**) Emission kinetics recorded during 30 min is represented as the average of five independent experiments in the presence of indicated reagents. The use of GTP or UTP as nucleotide substrate, MgCl_2_ as metal donor, or catalytically-inactive mutant NS5-GNN failed to render a measurable fluorescence signal. Experiments were carried out as described in Methods.

Preliminary real-time assays, involving the addition of SYBR Green II to the sample before initiating the reaction, showed an undetectable (using poly-C) or barely detectable (using poly-U) increase in fluorescence. In contrast to real-time experiments, we found significant increases in polymerase activity in an end-point experiment where the dye was added after the reaction was completed (Fig. S1). Previous studies have documented that an excess of SYBR Green I, chemically related to SYBR Green II, can inhibit other polymerase activities, such as those of Taq polymerase^43^ or human PrimPol^38^. Our resulted suggested that SYBR Green II acts as an inhibitor of ZIKV RdRp activity. Thus, we decided to test other fluorescent dyes for the real-time detection of newly synthesized dsRNA. It has been reported that SYTO 9 dye shows lower interference on polymerization assays when binding to dsDNA^44,45^. In contrast to the assays with SYBR Green II, we found that both end-point and real-time polymerization assays resulted in similar increases in fluorescence when using poly-U as template (Fig. S1, compare A with D). The relative increase in emitted fluorescence (ratio between the values obtained after a 60 min reaction and the background value observed at time 0) was similar using both approaches. This result suggested that SYTO 9 does not inhibit ZIKV RdRp, and thus can be used for real-time detection of activity. We also observed high reproducibility among different experimental samples, as reflected in the modest standard error values in different experiments (Fig. S1D). According to our assay, dsRNA synthesis was linear up to 60 min, and then reached the maximum accumulation of product at 150–180 min (Fig. 2A). ZIKV RdRp activity is dependent on the presence of Mn^2+^ in agreement with our data in radioactivity-based assays. The disruption of the catalytic site (RdRp GNN) also led to complete loss of polymerase activity (Fig. 2B). Likewise, terminal transferase activity was not detected in assays using poly-U as template and either GTP or UTP as substrate.

To further investigate the possible use of SYTO 9 dye to detect activity in the presence of other templates, we used poly-C. However, we detected increases in fluorescence only in end-point reactions, when SYTO 9 was added after the recation was complete, and not in a continuous reading assay when it was added before initiating the reaction (Fig. S1B). These results suggest that poly-C is not a suitable substrate for real-time assays.

### Optimization of the fluorescence-based assay

To improve the detection of RNA synthesis, we examined how changes in the concentration of reagents (i.e., NaCl, DTT, MnCl_2_ and enzyme) affected RdRp activity. The presence or absence of DTT and NaCl in the assay had little effect on RNA synthesis, which was only slightly impeded at high concentrations (Fig. S3A,B). As expected, no increase in fluorescence was detected when using MgCl_2_ (0 to 20 mM), whereas maximum activity was recorded with 2.5 mM MnCl_2_ (Fig. S3C). The increase in the velocity of reaction correlated linearly with increases in RdRp concentration along the 10–250 nM range. The maximum velocity was reached with 750 nM RdRp in the assay (Fig. S3D). From these assays we obtained a K_m_ for poly-U of 3.3 ± 0.5 μg/mL (~31 nM) and a K_m_ for ATP of 561 ± 38 μM (Fig. S4).

### Fluorescence-based activity can be inhibited by broad-range antiviral compounds

**Figure 3:**
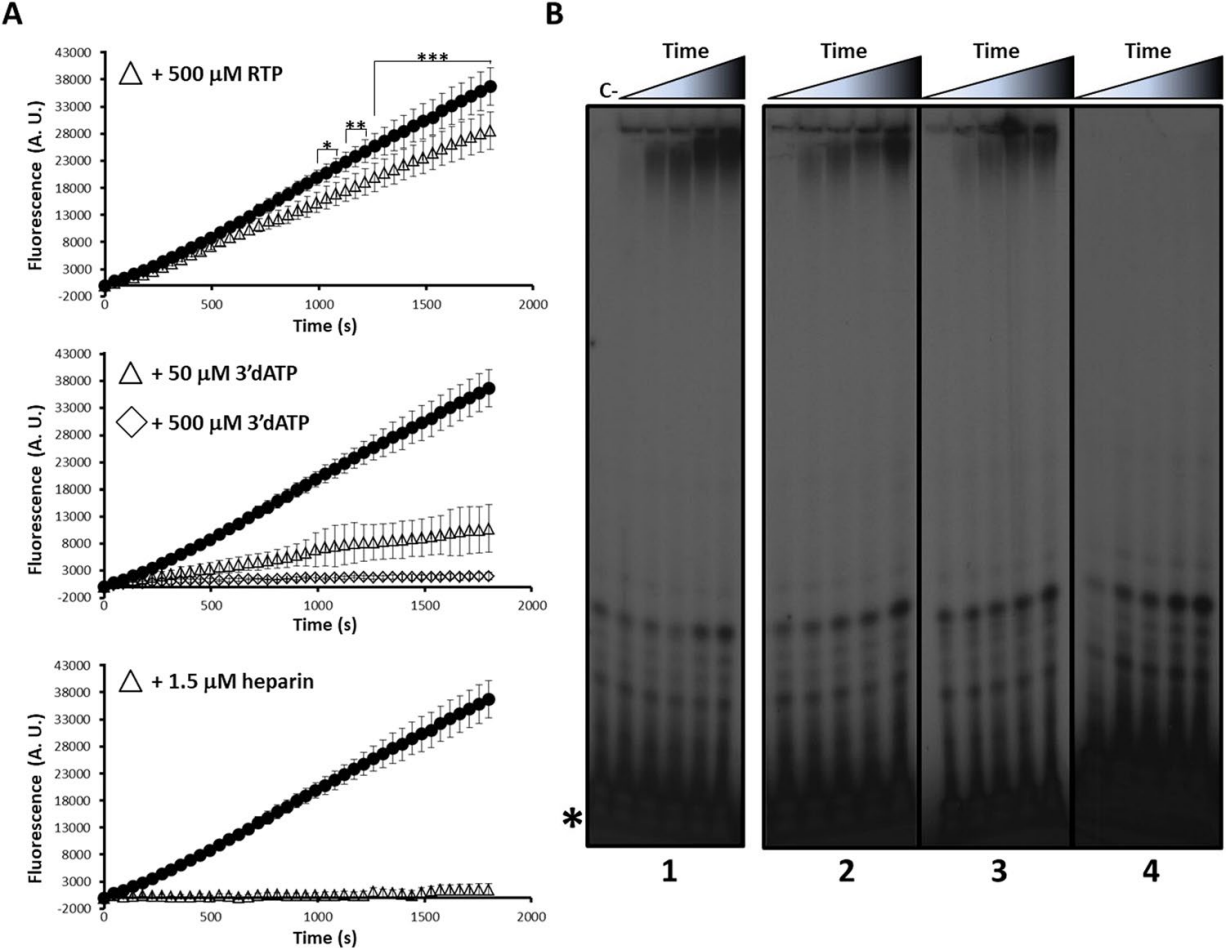
Enzymatic activity of ZIKV NS5 RdRp in the presence of inhibitory compounds. (**A**) Real-time fluorescence-based polymerization assays in the absence (black circles, same as in Fig. 2B), or in the presence of different inhibitory compounds at the indicated concentrations. Shown are average values obtained from five independent experiments. Statistically significant differences of fluorescence in the presence of RTP, relative to its absence, are represented (upper panel; *P < 0.01; **P < 0.005; ***P < 0.001). (**B**) Radioactive nucleotide assays using poly-U as a template molecule and [α-^32^P]ATP as the reaction substrate. Different inhibitory compounds were added to the reaction at the indicated concentrations. Elongation reactions were stopped at different time points (from 0 to 3 hours) in the presence of 500 μM RTP (panel 2), 50 μM 3′dATP (panel 3) or in the presence of 1.5 μM heparin (panel 4) and loaded onto 8 M urea-containing 20% polyacrylamide gels. The same electropherogram previously shown in Fig. 1 is displayed here (panel 1) for comparison. Representative gel images are shown. The panels were cropped from different full-length gels that were run and autoradiographied using the same experimental conditions. The position of the labeled non-incorporated nucleotide is indicated (*). See Methods for details.

We hypothesized that this fluorescence-based method could be exploited for the development of high-throughput screening methods to identify polymerase inhibitors. To test this, we used several broad-spectrum nucleoside and non-nucleoside polymerase inhibitors. Addition of polymerase NNI heparin^46,47^ to the reaction completely abrogated fluorescence-associated activity (Fig. 3A). To further confirm the sensitivity of our assay to inhibitors, we tested two nucleoside analogs: cordycepin 5′-triphosphate (3′dATP; a chain terminator analog of ATP^48–50^) and ribavirin 5′-triphosphate (RTP; a purine analog that inhibits but does not terminate RNA elongation during viral replication^51–54^). Both compounds reduced the polymerase activities (Fig. 3A). We calculated the IC_50_ values of these compounds (Fig. S3): as expected, the most potent inhibitor was the NNI heparin (IC_50_ = 81 ± 21 nM), followed by the NAIs, 3′dATP (54 ± 7 μM) and RTP (946 ± 46 μM). To confirm that the decrease in fluorescence was linked to the inhibition of RNA synthesis, we repeated these experiments using radioactive-labeled nucleotides. We found a reduced polymerization activity in the presence of inhibitors (3′dATP, RTP and heparin) that correlated with the aforementioned IC_50_ values (Fig. 3B). Similar inhibitory activities were observed when poly-C or poly-U were used as template molecules in radioactive-based activity assays (Fig. S2).

The robustness and suitability of this assay as a prospective, high-throughput method to screen polymerase antiviral compounds was examined by calculating the Z′ value, a standard statistical measure to evaluate the quality for high-throughput platforms^39^. The relative activity of both positive and negative controls was calculated as the average value obtained from 8 independent experiments (see Methods). Each experiment was carried out in triplicate and on independent days. Relative activity values were determined as the velocity of polymerization recorded during the first 10 min of the reaction. The mean Z’ value obtained was 0.62, which according to published standards, qualifies our method as an excellent assay for high-throughput screening application^39^.

### Fluorescence-based activity assay can be adapted to monitor different viral RdRps

**Figure 4:**
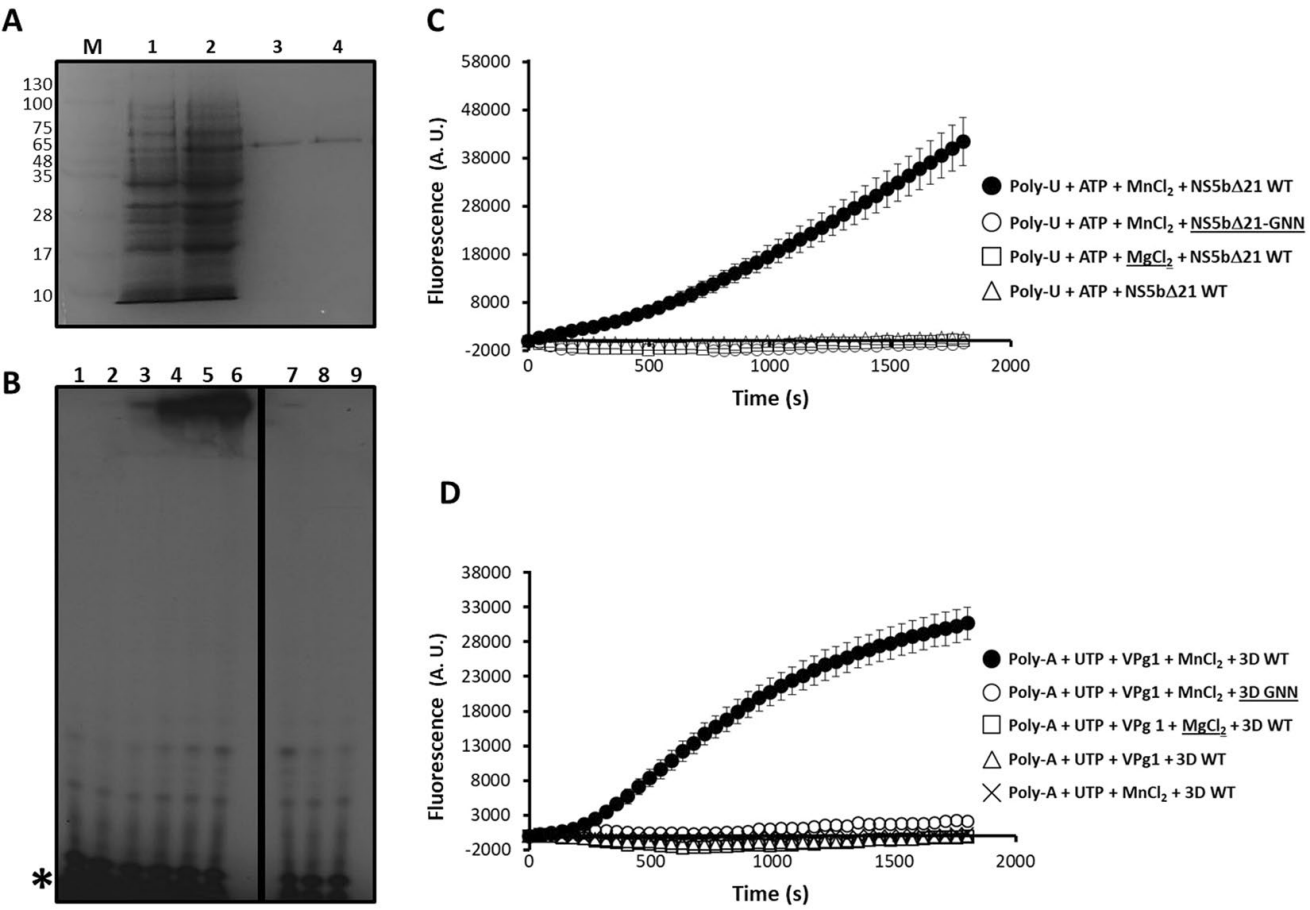
Real-time fluorescence-based polymerization assay using HCV RdRp and FMDV RdRp. (**A**) SDS-PAGE analysis of different purification stages of HCV NS5bΔ21 and HCV NS5bΔ21-GNN polymerases after expression in *E. coli*. Lane 1, a lysate of *E. coli* BL21(DE3)-pRIL containing plasmid pET28-HCV NS5bΔ21 and grown in the absence of IPTG is shown. Lane 2, same as lane 1 but grown in the presence of 500 μM IPTG. Purified proteins HCV NS5bΔ21 (lane 3) and HCV NS5bΔ21-GNN (lane 4) obtained after purification through Ni-NTA resin. M, molecular marker. The molecular weight of each band is indicated in kDa. (**B**) *De novo* polymerase activity performed by recombinant HCV RdRp in the presence of [α-^32^P]ATP using poly-U as template. Each lane represents a reaction stopped at a different time point (from 0 to 2 hours; lanes 2 to 6). Polymerization assays in the presence of MgCl_2_ (lane 7), in the absence of any metal donor (lane 8), or using GNN inactive mutant (lane 9) showed no detectable activity. Lane 1 represents a negative control reaction in the absence of enzyme. The position of the labeled non-incorporated nucleotide is indicated (*). A vertical black line separates non-contiguous images from the same gel. (**C**) Real-time determination of fluorescence emitted in assays containing purified NS5bΔ21 wild-type (WT) and NS5bΔ21 GNN, and in the presence of different components indicated in the figure. Shown are average values obtained from five different replicas. (**D**) Same as in (**C**) but using FMDV 3D WT and GNN RdRps. Gel electrophoresis, visualization by autoradiography analysis and fluorometric measurements were performed as described in Methods.

To investigate whether the assay can be adapted to other viral polymerases, we used two unrelated RdRps from FMDV (3Dpol) and HCV (recombinant NS5B) (Fig. 4A). We found that recombinant HCV polymerase can synthesize RNA *de novo* using radiolabeling (Fig. 4B) and the fluorescence-based approach (Fig. 4C), which is in agreement with the mechanism of genome replication for this virus^52,53^. Again, an increase in fluorescence was only observed with a catalytically-active HCV RdRp (NS5bΔ21) but not an inactive mutant, and the activity was dependent on the presence of Mn^2+^ (Fig. 4C). We also found that FMDV 3D polymerase can catalyze RNA synthesis *in vitro*, in an assay primed by the viral protein-primer VPg. It has been previously demonstrated that FMDV 3D catalyzes the addition of a uridine-monophosphate residue to Tyr3 in VPg. Once uridylylated, VPg can act as a competent primer to initiate viral genome replication^55,56^. Vpg protein-primed polymerization *in vitro* can be achieved using a polyadenylic acid as template, UTP as substrate, Mn^2+^ as catalytic metal and the VPg1 peptide synthetically produced^35^. Real-time experiments showed an increase of fluorescence as a function of time when using active 3D, whereas no activity was monitored in the presence of a catalytically-inactive protein, or in the absence of Mn^2+^ or VPg1 (Fig. 4D). Overall, our data show that this 96-well format assay can be exploited to characterize different viral RdRps *in vitro*, as it permits real-time monitoring of replication, allowing the characterization of small-molecule libraries in a cost-effective and rapid manner.

## Discussion

There is an urgent need to develop new treatments for ZIKV infection and to control its rapid geographical spread. Different approaches for the discovery of potential small-molecule inhibitors include the screening of chemical libraries, molecular modeling and virtual screening^29,57–59^. Although promising developments in this direction have been achieved (reviewed in^32^), there are as yet no antiviral agents licensed against ZIKV at the clinical level. Owing to significant differences in the mechanisms of replication between cellular DNA and viral RNA genomes, the latter involving the synthesis of RNA molecules templated by RNA, RdRps are attractive targets for the development of specific antiviral treatments. The use of antivirals against non-RdRp viral polymerases, such as human immunodeficiency virus and hepatitis B virus reverse transcriptases, and herpes virus DNA polymerase, supports the suitability of this group of enzymes as therapeutic targets^60^. Accordingly, the development of a fast and reproducible method for the screening of compounds with anti-ZIKV properties is a promising advance.

Several methods for high-throughput drug screening against virus RdPs have been described and validated^61–66^. However, there are several practical limitations to these approaches, such as the requirement for radioactive substances, entailing additional biosafety measures^63^, or an arduous experimental setup^65,66^ when compared with fluorescence-based methods^61,62,64^. Indeed, an advantage of fluorescence-based methods over traditional approaches is the absence of radioactive compounds, which facilitates their broad use in different laboratory settings without specific facilities or training requirements. An additional advantage of our strategy is that it allows the time-resolving determination of the polymerase activity, which we believe gives further insight into the mechanisms of replication and inhibition.

There have been previous attempts to develop high-throughput activity assays to identify drug inhibitors specifically against ZIKV RdRp. These methods include the use of either radioactive nucleotides^41^, or costly fluorescent-labeled RNA substrates^40^. Our method has the advantages of being a more economically affordable alternative, as it uses inexpensive homopolymeric RNA as a template substrate instead of labeled synthetic heteropolymeric RNA. Our approach would also allow for assay scale-up to high-throughput formats (e.g., 96 or 384-well formats, automation, etc.), for the rapid testing of small-molecule compound libraries^61,62,64^.

Fluorometric measurements of polymerase activity in the absence/presence of three representative inhibitors (heparin, 3′ATP and RTP) revealed a positive correlation with the results using a traditional assay based on radiolabeled nucleotides, further validating the fluorescence-based approach for the screening of antiviral compounds. The possibility of visualizing dsRNA synthesis in real-time increases the sensitivity of the assay, allowing an accurate determination of replication kinetics in the presence or absence of the drug and the predicted affinity constants of the compound tested. This method also permits the characterization of inhibitory molecules *in vitro*, which is of use in the identification of prospective antivirals. As part of our studies we have shown that RTP elicits a mild inhibition on ZIKV RdRp polymerization *in vitro*. We posit that the observed inhibition might be a consequence of reduced polymerase efficacy to elongate molecules where an RTP residue is incorporated^52–54,67^. RTP is not a chain terminator and its incorporation into the viral RNA has been linked to an increase in transition mutation rates *in vitro* and in cell culture for different RNA viruses, including ZIKV^68–71^.

Under different experimental conditions, including the use of different concentrations of SYBR Green II and SYTO 9, we found that poly-C RNA was not a suitable substrate for the detection of polymerase activity in real-time. Conversely, both poly-A and poly-U homopolymers were effective as template molecules in real-time assays. We hypothesize that the inhibition of polymerase activity is produced by the interaction of RdRp with the dye during dsRNA synthesis. It is possible that poly-G synthesis as a result of replicating poly-C can lead to non-canonical G-quadruplex structures^72^, which in the presence of a fluorophore can further increase their inherently elevated stability^44^. Thus, these RNA-dye complexes might impede the effective elongation by the viral RdRp.

In conclusion, we have demonstrated that the procedures developed here can be easily adapted to measure polymerization activity of several viral RdRps, strengthening our method as a universal procedure for the development of high-throughput tools to characterize viral polymerases (e.g., enzymology, polymerase variants of interest) and to screen small-molecule libraries to identify antiviral drugs. In particular, we believe that this platform can be a useful tool for the development of therapeutics against ZIKV and other flaviviruses, which are currently unavailable.

## Supplementary information

Supplementary Information

## References

[1] Kuno G, Chang GJ, Tsuchiya KR, Karabatsos N, Cropp CB (1998). Phylogeny of the genus Flavivirus. J Virol.

[2] Weaver SC (2016). Zika virus: History, emergence, biology, and prospects for control. Antiviral Res.

[3] Besnard, M., Lastere, S., Teissier, A., Cao-Lormeau, V. & Musso, D. Evidence of perinatal transmission of Zika virus, French Polynesia, December 2013 and February 2014. *Euro Surveill* **19**, 10.2807/1560-7917.ES2014.19.13.20751 (2014). 24721538

[4] Musso D (2015). Potential sexual transmission of Zika virus. Emerg Infect Dis.

[5] Musso, D. *et al*. Potential for Zika virus transmission through blood transfusion demonstrated during an outbreak in French Polynesia, November 2013 to February 2014. *Euro Surveill* **19**, 10.2807/1560-7917.ES2014.19.14.20761 (2014). 10.2807/1560-7917.es2014.19.14.20761 24739982

[6] Petersen LR, Jamieson DJ, Powers AM, Honein MA (2016). Zika Virus. N Engl J Med.

[7] Lazear HM, Diamond MS (2016). Zika Virus: New Clinical Syndromes and Its Emergence in the Western Hemisphere. J Virol.

[8] Mlakar J (2016). Zika Virus Associated with Microcephaly. N Engl J Med.

[9] Rasmussen SA, Jamieson DJ, Honein MA, Petersen LR (2016). Zika Virus and Birth Defects–Reviewing the Evidence for Causality. N Engl J Med.

[10] Cao-Lormeau VM (2016). Guillain-Barre Syndrome outbreak associated with Zika virus infection in French Polynesia: a case-control study. Lancet.

[11] Gulland A (2016). Zika virus is a global public health emergency, declares WHO. BMJ.

[12] Kuno G, Chang GJ (2007). Full-length sequencing and genomic characterization of Bagaza, Kedougou, and Zika viruses. Arch Virol.

[13] Yun, S. I. *et al*. Complete Genome Sequences of Three Historically Important, Spatiotemporally Distinct, and Genetically Divergent Strains of Zika Virus: MR-766, P6-740, and PRVABC-59. *Genome Announc* **4**, 10.1128/genomeA.00800-16 (2016). 10.1128/genomeA.00800-16 PMC4991703 27540058

[14] Ladner, J. T. *et al*. Complete Genome Sequences of Five Zika Virus Isolates. *Genome Announc* **4**, 10.1128/genomeA.00377-16 (2016). 10.1128/genomeA.00377-16 PMC4866861 27174284

[15] Lindenbach BD, Rice CM (2003). Molecular biology of flaviviruses. Adv Virus Res.

[16] Zhao B (2017). Structure and function of the Zika virus full-length NS5 protein. Nat Commun.

[17] Wang B (2017). The structure of Zika virus NS5 reveals a conserved domain conformation. Nat Commun.

[18] Godoy AS (2017). Crystal structure of Zika virus NS5 RNA-dependent RNA polymerase. Nat Commun.

[19] Wu J, Liu W, Gong P (2015). A Structural Overview of RNA-Dependent RNA Polymerases from the Flaviviridae Family. Int J Mol Sci.

[20] Steitz TA, Steitz JA (1993). A general two-metal-ion mechanism for catalytic RNA. Proc Natl Acad Sci USA.

[21] Wassenegger M, Krczal G (2006). Nomenclature and functions of RNA-directed RNA polymerases. Trends Plant Sci.

[22] Yin Z (2009). An adenosine nucleoside inhibitor of dengue virus. Proc Natl Acad Sci USA.

[23] Gotte M, Feld JJ (2016). Direct-acting antiviral agents for hepatitis C: structural and mechanistic insights. Nat Rev Gastroenterol Hepatol.

[24] Malet H (2008). The flavivirus polymerase as a target for drug discovery. Antiviral Res.

[25] Lim SP, Noble CG, Shi PY (2015). The dengue virus NS5 protein as a target for drug discovery. Antiviral Res.

[26] Eyer L (2015). Nucleoside inhibitors of tick-borne encephalitis virus. Antimicrob Agents Chemother.

[27] Chen H (2013). Selective inhibition of the West Nile virus methyltransferase by nucleoside analogs. Antiviral Res.

[28] Julander JG (2010). Efficacy of 2′-C-methylcytidine against yellow fever virus in cell culture and in a hamster model. Antiviral Res.

[29] Sebera J (2018). The structural model of Zika virus RNA-dependent RNA polymerase in complex with RNA for rational design of novel nucleotide inhibitors. Sci Rep.

[30] Keating GM, Vaidya A (2014). Sofosbuvir: first global approval. Drugs.

[31] Saiz, J. C. & Martin-Acebes, M. A. The Race To Find Antivirals for Zika Virus. *Antimicrob Agents Chemother* **61**, 10.1128/AAC.00411-17 (2017). 10.1128/AAC.00411-17 PMC5444186 28348160

[32] Wang B, Thurmond S, Hai R, Song J (2018). Structure and function of Zika virus NS5 protein: perspectives for drug design. Cell Mol Life Sci.

[33] Basile K, Kok J, Dwyer DE (2017). Zika virus: what, where from and where to?. Pathology.

[34] Sambrook, J. & Green, M. R. *Molecular cloning. A Laboratory Manual*. 4th edn, (Cold Spring Harbor Laboratory Press, 2012).

[35] Arias A (2005). Mutant viral polymerase in the transition of virus to error catastrophe identifies a critical site for RNA binding. J Mol Biol.

[36] Ferrer-Orta C (2004). Structure of foot-and-mouth disease virus RNA-dependent RNA polymerase and its complex with a template-primer RNA. J Biol Chem.

[37] Marukian S (2008). Cell culture-produced hepatitis C virus does not infect peripheral blood mononuclear cells. Hepatology.

[38] Agudo R, Calvo PA, Martinez-Jimenez MI, Blanco L (2017). Engineering human PrimPol into an efficient RNA-dependent-DNA primase/polymerase. Nucleic Acids Res.

[39] Zhang JH, Chung TD, Oldenburg KR (1999). A Simple Statistical Parameter for Use in Evaluation and Validation of High Throughput Screening Assays. J Biomol Screen.

[40] Lu, G. *et al*. Analysis of Ribonucleotide 5′-Triphosphate Analogs as Potential Inhibitors of Zika Virus RNA-Dependent RNA Polymerase by Using Nonradioactive Polymerase Assays. *Antimicrob Agents Chemother* **61**, 10.1128/AAC.01967-16 (2017). 10.1128/AAC.01967-16 PMC5328514 27993851

[41] Xu HT (2017). Purification of Zika virus RNA-dependent RNA polymerase and its use to identify small-molecule Zika inhibitors. J Antimicrob Chemother.

[42] Calmels C, Ventura M, Aknin C, Metifiot M, Andreola ML (2017). De novo RNA synthesis catalyzed by the Zika Virus RNA polymerase domain. Sci Rep.

[43] Driscoll MD, Rentergent J, Hay S (2014). A quantitative fluorescence-based steady-state assay of DNA polymerase. FEBS J.

[44] Gudnason H, Dufva M, Bang DD, Wolff A (2007). Comparison of multiple DNA dyes for real-time PCR: effects of dye concentration and sequence composition on DNA amplification and melting temperature. Nucleic Acids Res.

[45] Monis PT, Giglio S, Saint CP (2005). Comparison of SYTO9 and SYBR Green I for real-time polymerase chain reaction and investigation of the effect of dye concentration on amplification and DNA melting curve analysis. Anal Biochem.

[46] Bai X, Fischer S, Keshavjee S, Liu M (2000). Heparin interference with reverse transcriptase polymerase chain reaction of RNA extracted from lungs after ischemia-reperfusion. Transpl Int.

[47] Beutler E, Gelbart T, Kuhl W (1990). Interference of heparin with the polymerase chain reaction. Biotechniques.

[48] Izuta S, Kohsaka-Ichikawa M, Yamaguchi T, Saneyoshi M (1996). 3′-Deoxyribonucleotides inhibit eukaryotic DNA primase. J Biochem.

[49] Arnold JJ, Smidansky ED, Moustafa IM, Cameron CE (2012). Human mitochondrial RNA polymerase: structure-function, mechanism and inhibition. Biochim Biophys Acta.

[50] Rose KM, Bell LE, Jacob ST (1977). Specific inhibition of chromatin-associated poly(A) synthesis *in vitro* by cordycepin 5′-triphosphate. Nature.

[51] Sierra M (2007). Foot-and-mouth disease virus mutant with decreased sensitivity to ribavirin: implications for error catastrophe. J Virol.

[52] Maag D, Castro C, Hong Z, Cameron CE (2001). Hepatitis C virus RNA-dependent RNA polymerase (NS5B) as a mediator of the antiviral activity of ribavirin. J Biol Chem.

[53] Crotty S (2000). The broad-spectrum antiviral ribonucleoside ribavirin is an RNA virus mutagen. Nat Med.

[54] Arias A (2008). Determinants of RNA-dependent RNA polymerase (in)fidelity revealed by kinetic analysis of the polymerase encoded by a foot-and-mouth disease virus mutant with reduced sensitivity to ribavirin. J Virol.

[55] Nayak A, Goodfellow IG, Belsham GJ (2005). Factors required for the Uridylylation of the foot-and-mouth disease virus 3B1, 3B2, and 3B3 peptides by the RNA-dependent RNA polymerase (3Dpol) *in vitro*. J Virol.

[56] Nayak A (2006). Role of RNA structure and RNA binding activity of foot-and-mouth disease virus 3C protein in VPg uridylylation and virus replication. J Virol.

[57] Eyer L (2016). Nucleoside Inhibitors of Zika Virus. J Infect Dis.

[58] Ramharack P, Soliman MES (2018). Zika virus NS5 protein potential inhibitors: an enhanced in silico approach in drug discovery. J Biomol Struct Dyn.

[59] Hercik K (2017). Adenosine triphosphate analogs can efficiently inhibit the Zika virus RNA-dependent RNA polymerase. Antiviral Res.

[60] De Clercq E (2005). Recent highlights in the development of new antiviral drugs. Curr Opin Microbiol.

[61] Eltahla AA, Lackovic K, Marquis C, Eden JS, White PA (2013). A fluorescence-based high-throughput screen to identify small compound inhibitors of the genotype 3a hepatitis C virus RNA polymerase. J Biomol Screen.

[62] Niyomrattanakit P (2011). A fluorescence-based alkaline phosphatase-coupled polymerase assay for identification of inhibitors of dengue virus RNA-dependent RNA polymerase. J Biomol Screen.

[63] Gong EY (2013). Expression and purification of dengue virus NS5 polymerase and development of a high-throughput enzymatic assay for screening inhibitors of dengue polymerase. Methods Mol Biol.

[64] Campagnola G, Gong P, Peersen OB (2011). High-throughput screening identification of poliovirus RNA-dependent RNA polymerase inhibitors. Antiviral Res.

[65] Su CY (2010). High-throughput identification of compounds targeting influenza RNA-dependent RNA polymerase activity. Proc Natl Acad Sci USA.

[66] Madhvi A (2017). A screen for novel hepatitis C virus RdRp inhibitor identifies a broad-spectrum antiviral compound. Sci Rep.

[67] Vo NV, Young KC, Lai MM (2003). Mutagenic and inhibitory effects of ribavirin on hepatitis C virus RNA polymerase. Biochemistry.

[68] Bassi, M. R., Sempere, R. N., Meyn, P., Polacek, C. & Arias, A. Extinction of Zika virus and Usutu virus by lethal mutagenesis reveals different patterns of sensitivity to three mutagenic drugs. *Antimicrob Agents Chemother*, 10.1128/AAC.00380-18 (2018). 10.1128/AAC.00380-18 PMC6125542 29914957

[69] Crotty S, Cameron CE, Andino R (2001). RNA virus error catastrophe: direct molecular test by using ribavirin. Proc Natl Acad Sci USA.

[70] Graci JD, Cameron CE (2008). Therapeutically targeting RNA viruses via lethal mutagenesis. Future Virol.

[71] Bonnac LF, Mansky LM, Patterson SE (2013). Structure-activity relationships and design of viral mutagens and application to lethal mutagenesis. J Med Chem.

[72] Fay MM, Lyons SM, Ivanov P (2017). RNA G-Quadruplexes in Biology: Principles and Molecular Mechanisms. J Mol Biol.

