# Supplementary material for: Development of a fluorescence-based method for the rapid determination of Zika virus polymerase activity and the screening of antiviral drugs
Source: Sci Rep. 2019 Apr 1;9:5397. doi: 10.1038/s41598-019-41998-1 (PMC6444013; doi:10.1038/s41598-019-41998-1)

Supplementary Information

Development of a fluorescence-based method for the rapid determination of Zika virus polymerase activity and the screening of antiviral drugs

Yanira Sáez-Álvarez[1], Armando Arias[2], Carmen del Aguila[1], and Rubén Agudo[1,*]

[1] Facultad de Farmacia, Universidad CEU San Pablo, Campus Montepríncipe, Boadilla del Monte 28668 Madrid, Spain.

[2] Technical University of Denmark, National Veterinary Institute, Kemitorvet, DK-2800, Kongens Lyngby, Denmark

Corresponding: ruben.agudotorres@ceu.es

Table S1. Primers used for cloning and directed mutagenesis

**Name**	**Sequence (5'→3')**
pET16_Fw	ctcgaggatccggctgctaacaaagccg
pET16_rv	catatgcgaccttcgatatggccgctgctg
pET28a_Fw	catggtatatctccttcttaaagttaaacaaaattatttc
pET28a_rv	ctcgagcaccaccaccaccaccactgagatc
NS5 short_pET16_Fw	agcagcggccatatcgaaggtcgtcatatgaagatcattggtaaccgcattgaaaggatc
NS5_pET16_rv	ttcgggctttgttagcagccggatcctcgagttattacagcactccaggtgtagacccttcttcac
NS5B_HCV_Fw	ttttgtttaactttaagaaggagatataccatgtccatgtcatactcctggaccggggctc
NS5B_Δ21_HCV_rv	atctcagtggtggtggtggtggtgctcgaggcggggtcgggcgcgcgacacgctgtg
NS5_GNN_Fw	gcagtcagtggaAATAATtgcgttgtgaagccaattg[1]
NS5_GNN_rv	ttcacaacgcaATTATTtccactgactgccattcgtttg[1]

[1] Capital letters indicate the codon modified by site-directed mutagenesis.

Figure S1. Comparison between end-point and real-time fluorescence-based polymerization assays using poly-C and poly-U as templates. (A–C) End-point fluorescence-based polymerization assays with ZIKV NS5 RdRp WT and GNN. Reactions were carried out in the absence (black bars) or presence (white bars) of 25 mM EDTA, using 500 μM ATP and poly-U (A), or 500 μM GTP and poly-C (in B and C), as template and substrate, respectively. Stop solution used in A and C contained 4× SYBR® Green II and 25 mM EDTA. Stop solution used in B contained 0.25 μM SYTO 9 and 25 mM EDTA. See Methods section for further details. Fluorescence emitted was recorded after stabilization of the samples in the presence of the dye (5 minutes incubation following mixing with the dye). The average of four independent experiments is represented. Statistically significant differences between the fluorescence recorded in the presence, or in the absence of EDTA, are represented (*P<0.01; ***P < 0.001). (D) Real-time fluorescence kinetics recorded during 1 h. Average values obtained from 4 independent standard reactions are represented, as indicated in Methods. The background fluorescence is not subtracted in this representation. The ratio of maximum fluorescence to background fluorescence emission recorded for ZIKV NS5 WT either in end-point or real-time approaches (A and D, respectively) are shown.

Figure S2. Drug inhibition of ZIKV RdRp activity in assays using poly-C as template. (A) Radiolabeled assay using poly-C as template. Left panel shows representative gel results of polymerization reactions performed with ZIKV RdRp in the presence poly-C and [α-^{32}P]GTP, and in the absence or presence of different inhibitory compounds. Elongation reactions were stopped at different time points (from 0 to 3 h) in the absence of drug (panel "No drug") or in the presence of 500 μM RTP (panel "+ 500 μM RTP") or 1.5 μM heparin

(panel "+ 1.5 μM RTP"). Polymerization assays in the absence of enzyme (lane 1), in the presence of $MgCl_2$ as metal donor (lane 2), in the absence of any metal donor (lane 3), or using inactive ZIKV RdRp (GNN mutant, lane 4) are shown. The panels were cropped from different full-length gels, which were run and subjected to phosphorimaging using similar experimental conditions. See experimental details in Methods. Right panel shows a phosphorimage from a reaction performed in the presence of poly-U and [α-^{32}P]ATP included for comparison. Note that this image has been obtained from the same gel shown in Figure 1B, Results section. (B) Radiolabeled control experiments to determine the possible terminal transferase activity of ZIKV RdRp either in the presence of poly-U and [α-^{32}P]CTP (lane 1) or poly-C and [α-^{32}P]ATP (lane 2). The position of the labeled non-incorporated nucleotide is indicated (*). Experimental protocols are explained in Methods.

Figure S3. Characterization of biochemical conditions for the fluorescence-based assay. The effect of variations in the concentration of different reagents in the assay was examined: (A) NaCl (B) DTT (C) $MgCl_2$ or $MnCl_2$ and (D) RdRp. Graphical representations showing the average of triplicate experiments. In each case, the activity shown is relative to the highest value of fluorescence detected in each assay, which was represented as 100%. In the case of an assay in the presence of $MgCl_2$, the activity was relative to a standard reaction in the presence of 2.5 mM $MnCl_2$ (see Methods).

Figure S4. Steady-state kinetics of ZIKV RdRp using a fluorescence-based method. The kinetics parameters of the reaction K_m and V_{max} were calculated for (A) the template poly-U, and (B) the substrate ATP. The experimental procedure for the calculation of these constants is described in Methods. Each value is obtained from at least three independent replicates.

Figure S5. IC_{50} determination of reference ZIKV RdRp inhibitors. IC_{50} values for (A) heparin (B) 3'dATP and (C) RTP were obtained. Each value is relative to the activity detected in the absence of inhibitor and is the average of at least three independent replicates.

Figure S6. Full-length gel pictures that were used to produce Figure 1B. (A) A black rectangle area corresponds to the cropped portion from this gel that appears in the left panel of Figure 1B. (B) A black rectangle area corresponds to the cropped portion from this gel (lanes 1, 2 and 3) that appears in the right panel of Figure 1B (lanes 7, 8 and 9, respectively). Lanes 2 and 3 correspond to lanes 8 and 9 in Figure 1B. The empty lane in this rectangle (lane"-") has been removed from the image in Figure 1B. Lane 4 represents a standard assay used as positive control that has not been included in Figure 1B. The position of non-incorporated nucleotides is indicated (*). Additional lanes on this gel unrelated to the study have been omitted.

Figure S7. Full-length gels used to produce Figure 3B. Panel 1 from Figure 3B is the same as the one represented in the left panel of Figure 1B (See legend of Figure 3). (A) A black rectangle corresponds to the cropped portion from this gel that appears in the panel 2 of Figure 3B. Lane 1 is a negative control in the absence of enzyme. This lane is not included in Figure 2B since this control already appears in Panel 1 (indicated there as "C-"). (B) A black rectangle corresponds to the cropped portion from this full-length gel that appears in the panel 3 of Figure 3B. Bands unrelated to the study have been removed from the full-length gel. (C) A black rectangle corresponds to the cropped portion from this gel that appears in panel 4 of Figure 3B. This part of the gel has also been included in the composition of Figure 1B. Note that the lanes inside the black rectangle have been flipped

horizontally to compose panel 4 of Figure 3B, to be consistent with the other panels shown in that figure. The position of non-incorporated nucleotides is indicated (*). Bands unrelated to the study have been removed from the gel.

Figure S8. Full-length gel used to produce the cropped gels of Figure 4B. Black rectangle (encompassing lanes 1 to 6) corresponds to the cropped portion from this gel used to compose the left part of Figure 4B (lanes 1 to 6, respectively). A red rectangle (encompassing lanes 7 to 9) corresponds to the cropped portion from this full-length gel that has been used to compose the right part of Figure 4B (lanes 7 to 9, respectively). The position of non-incorporated nucleotides is indicated (*).

Figure S9. Full-length gels used to produce Figure S2. (A) A black rectangle corresponds to the cropped portion from this gel that appears in the left panel of Figure S2A. This is exactly the same gel used to obtain the panel 1 of Figure 1B (see legend of Figure S3). In this case, the image was not obtained by autoradiography, but by phosphorimaging. Additional lanes corresponding to experiments not related to this study have been omitted. (B) A black rectangle corresponds to the cropped portion from this gel that appears in the panel “No drug” of Figure S2A. Bands unrelated to the study have been removed from the full-length gel. (C) A black rectangle corresponds to the cropped portion from this gel that then appears in the panel “+ 500 μM RTP” of Figure S2A. A red rectangle corresponds to the cropped potion from this full-length gel that appears in the panel “+ 1.5 μM heparin” of Figure S2A. A blue rectangle (encompassing lanes 1 and 2) corresponds to the cropped portion from this gel used to compose Figure S2B (lanes 1 and 2, respectively). This image was flipped horizontally in the final Figure S2B. (D) A black rectangle (encompassing lanes 1 to 4) corresponds to the cropped portion from this gel that appears in the first panel

of figure S2A counting from the right side (lanes 1 to 4, respectively). The position of non-incorporated nucleotides is indicated (*). Bands unrelated to the study have been removed from the full-length gel.

Figure S1

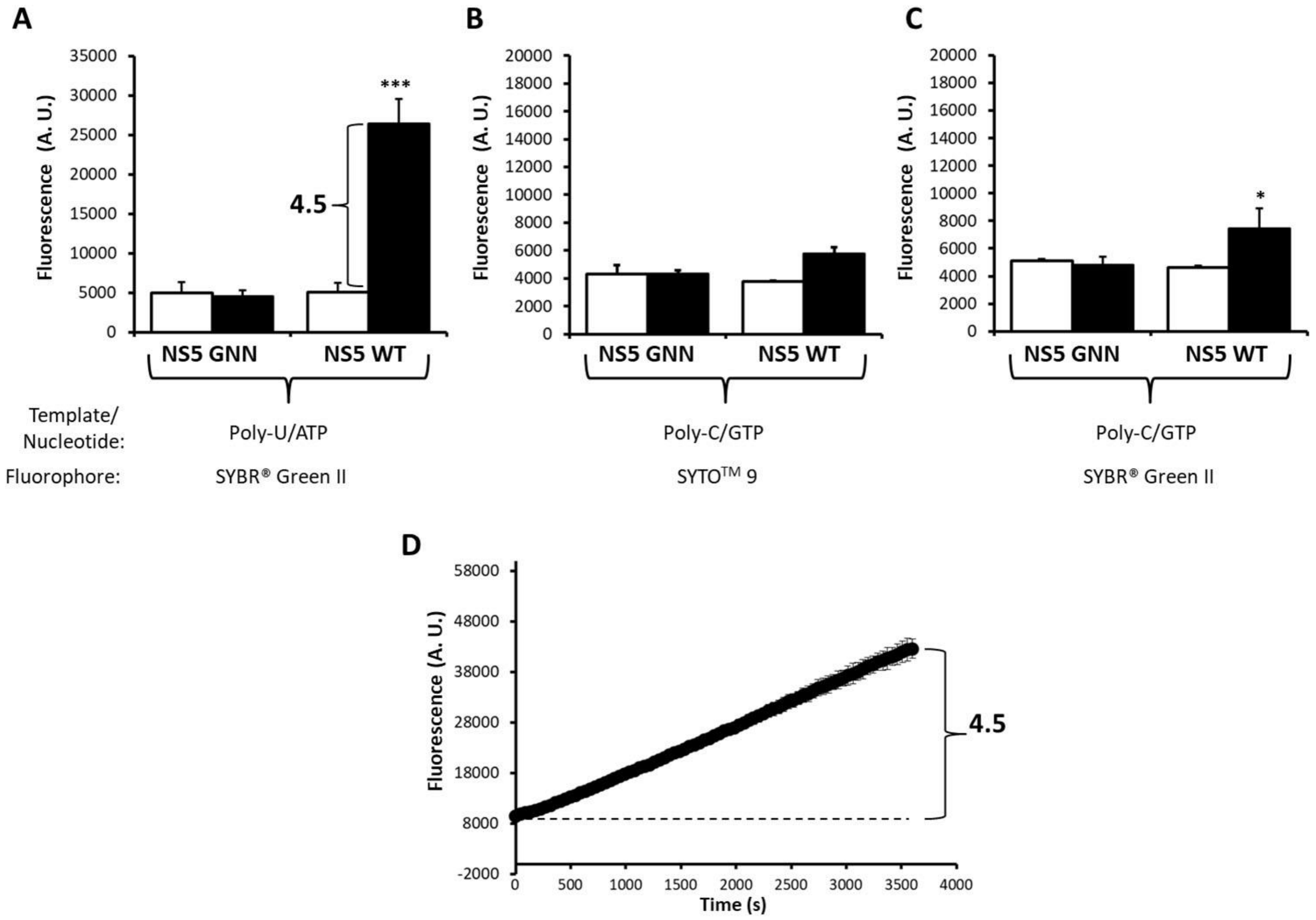
A
B
C
D
Fluorescence (A. U.)
4.5
***
*
NS5 GNN
NS5 WT
Template/
Nucleotide:
Fluorophore:
Poly-U/ATP
SYBR® Green II
Poly-C/GTP
SYTO™ 9
Poly-C/GTP
SYBR® Green II
Time (s)

Figure S2

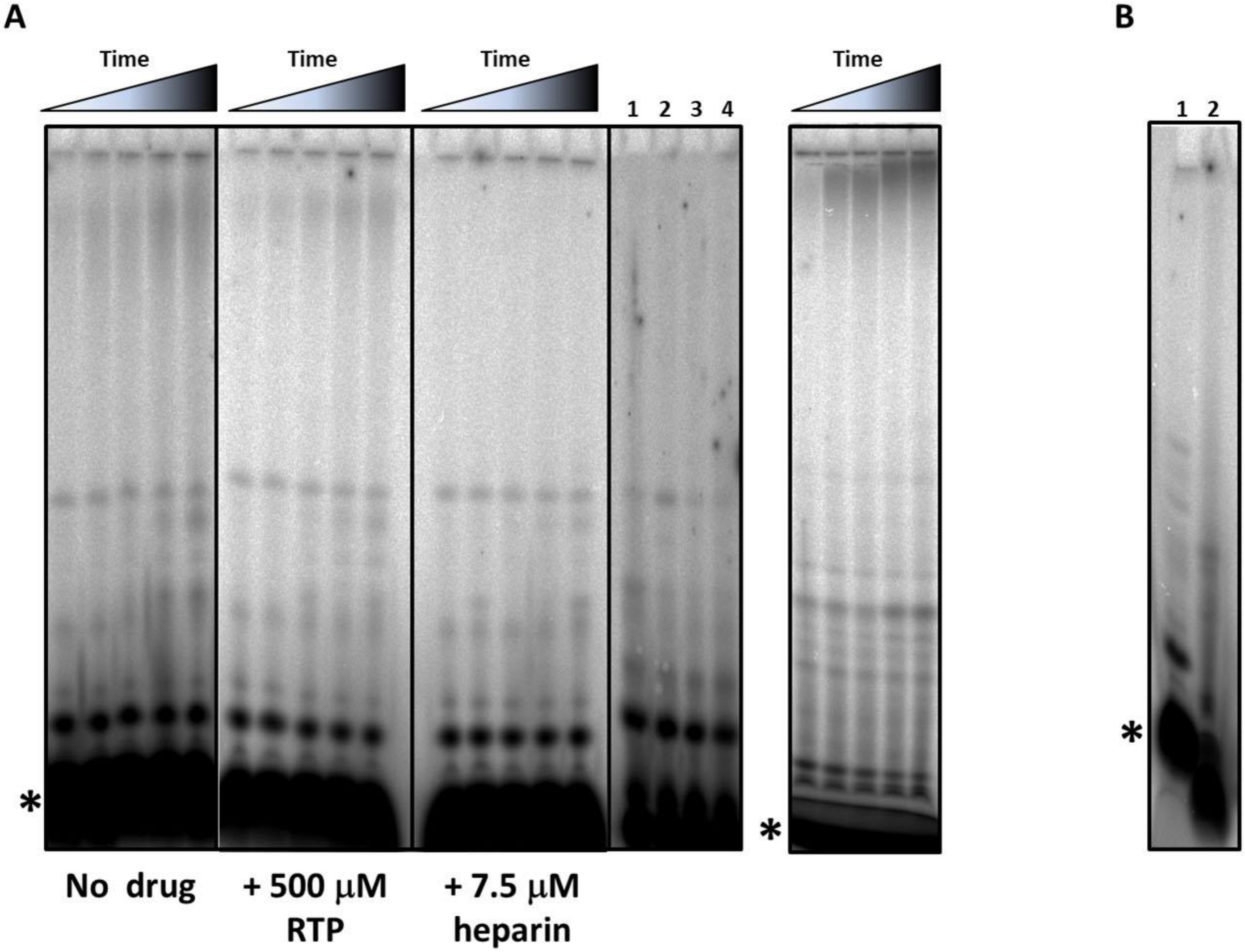

Figure S3

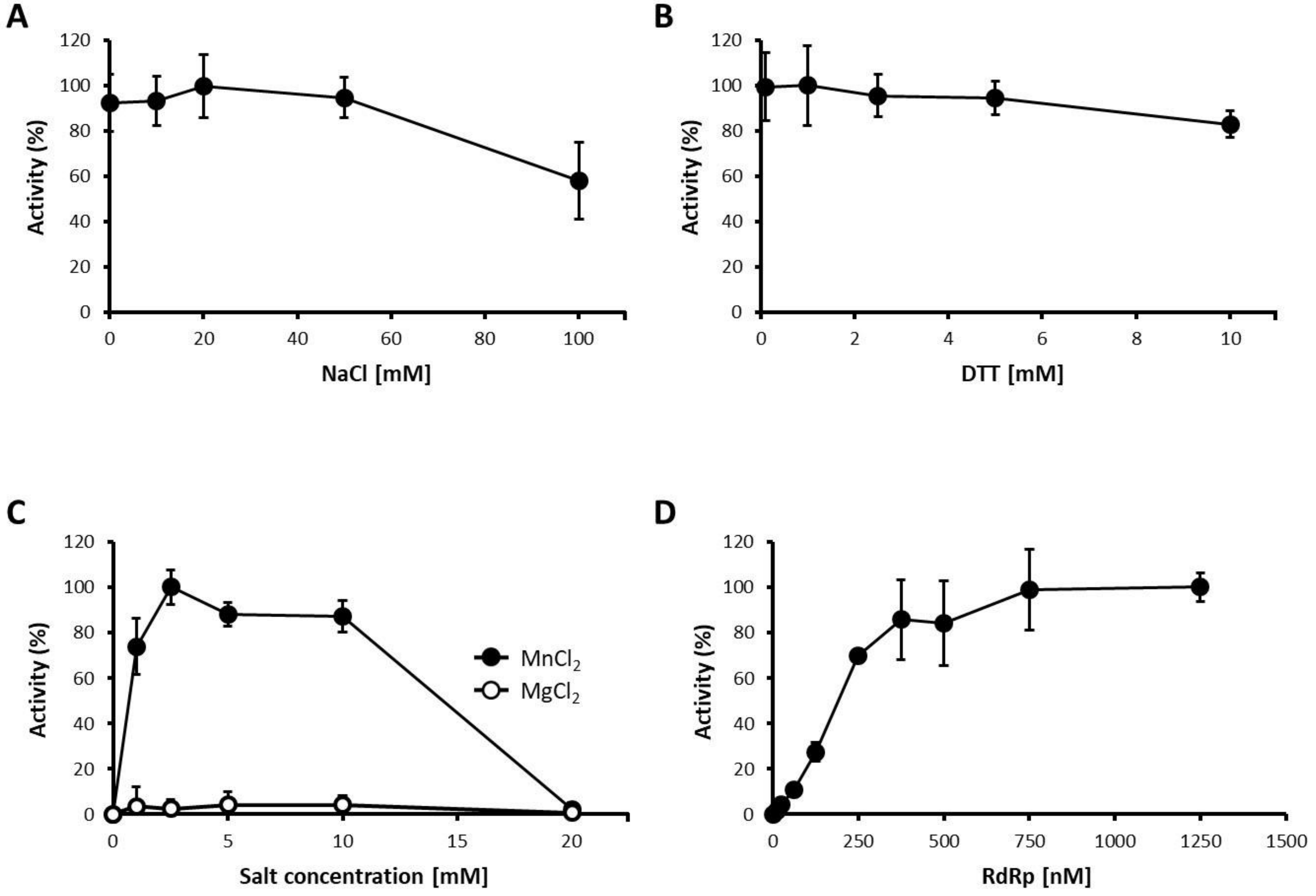

A
B
C
D
Activity (%)
NaCl [mM]
DTT [mM]
Salt concentration [mM]
RdRp [nM]
MnCl2
MgCl2
120
100
80
60
40
20
0
0
20
40
60
80
100
0
2
4
6
8
10
0
5
10
15
20
0
250
500
750
1000
1250
1500

Figure S4

A

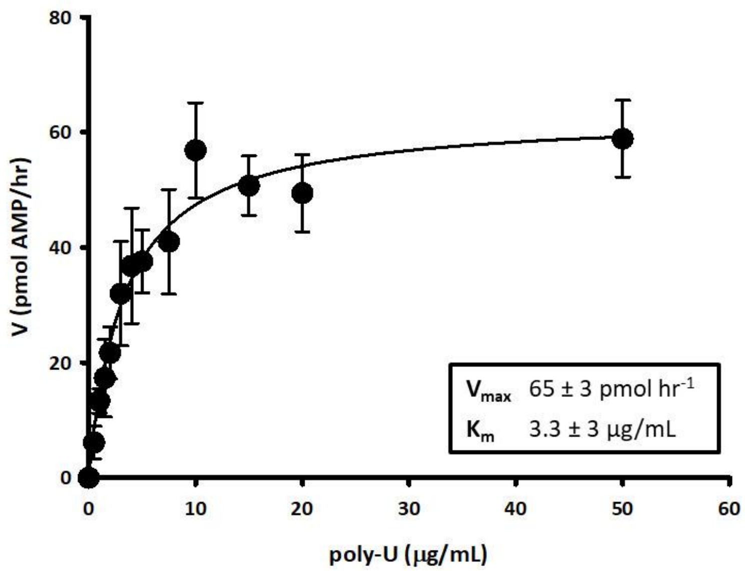

80
60
40
20
0
V (pmol AMP/hr)
0
10
20
30
40
50
60
poly-U (μg/mL)
Vmax 65 ± 3 pmol hr-1
Km 3.3 ± 3 μg/mL

B

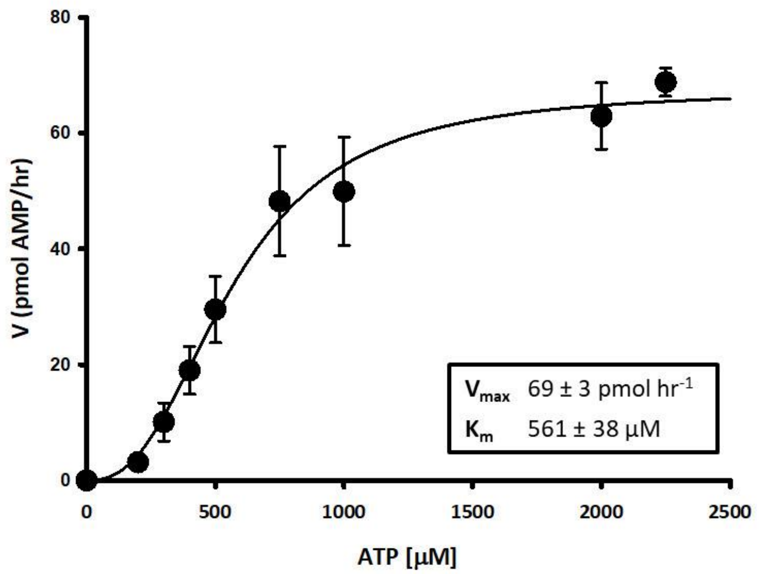

80
60
40
20
0
V (pmol AMP/hr)
0
500
1000
1500
2000
2500
ATP [μM]
Vmax 69 ± 3 pmol hr-1
Km 561 ± 38 μM

Figure S5

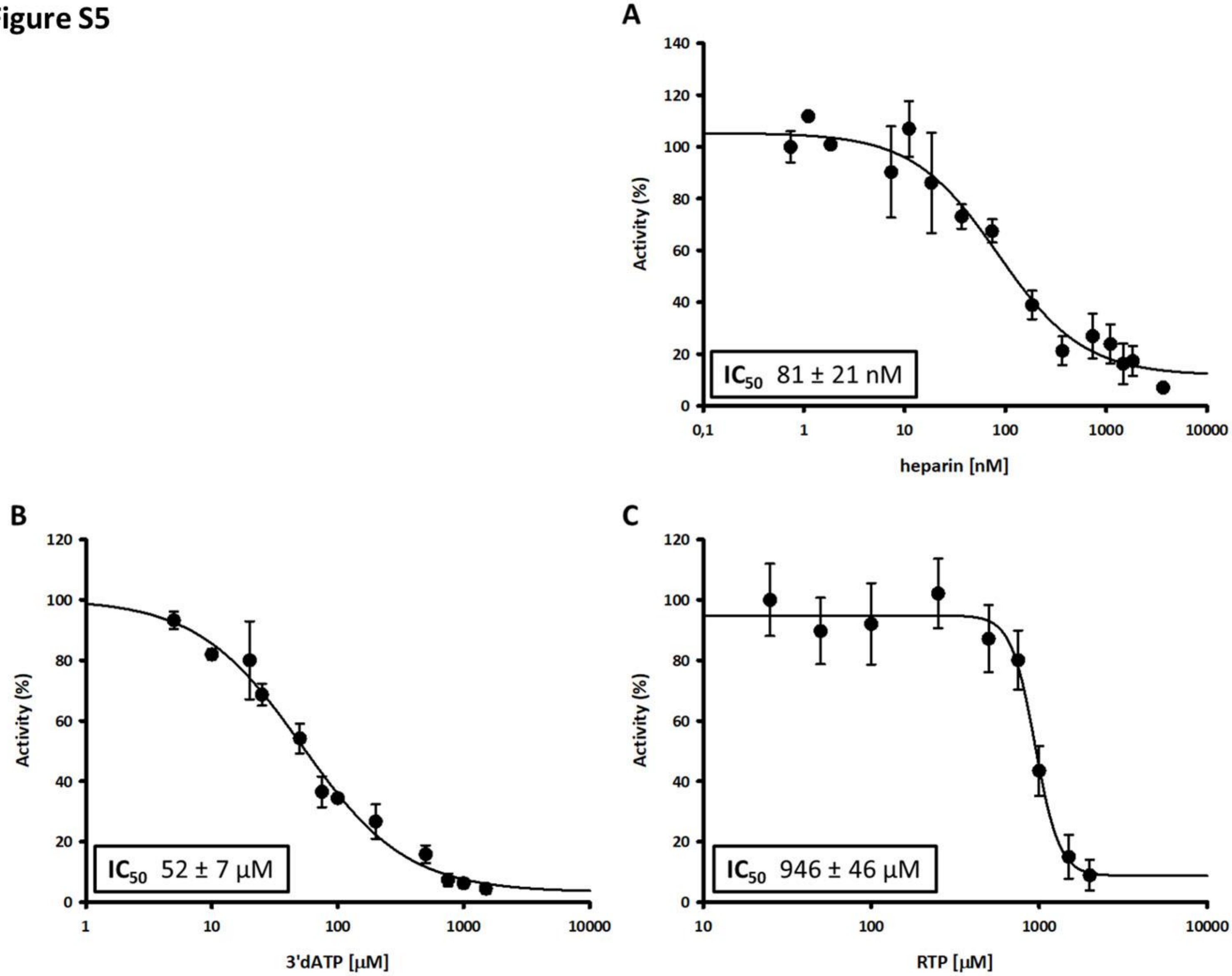
A
Activity (%)
140
120
100
80
60
40
20
0
0,1
1
10
100
1000
10000
heparin [nM]
IC50 81 ± 21 nM
B
Activity (%)
120
100
80
60
40
20
0
1
10
100
1000
10000
3'dATP [µM]
IC50 52 ± 7 µM
C
Activity (%)
120
100
80
60
40
20
0
10
100
1000
10000
RTP [µM]
IC50 946 ± 46 µM

Figure S6

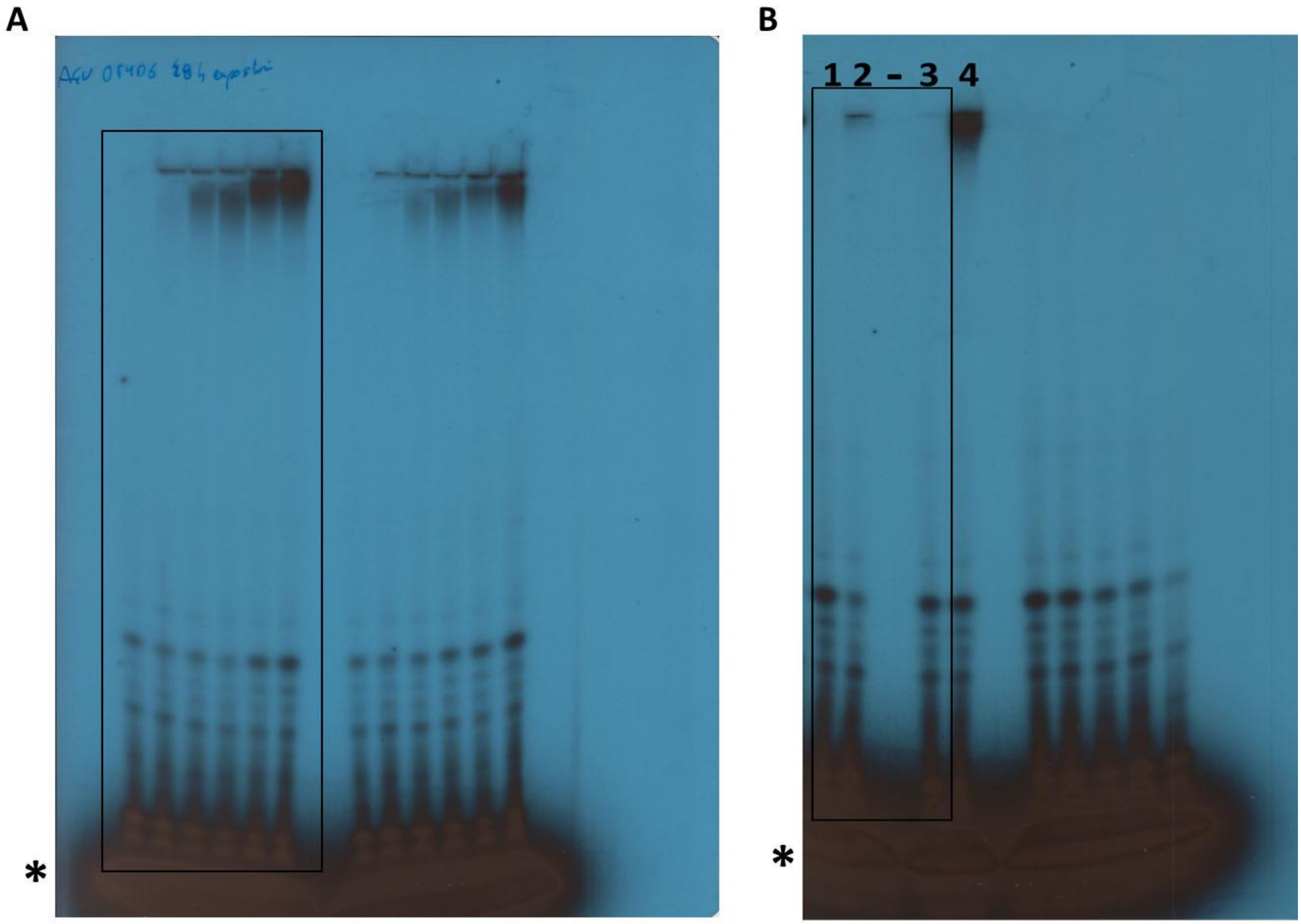

Figure S7

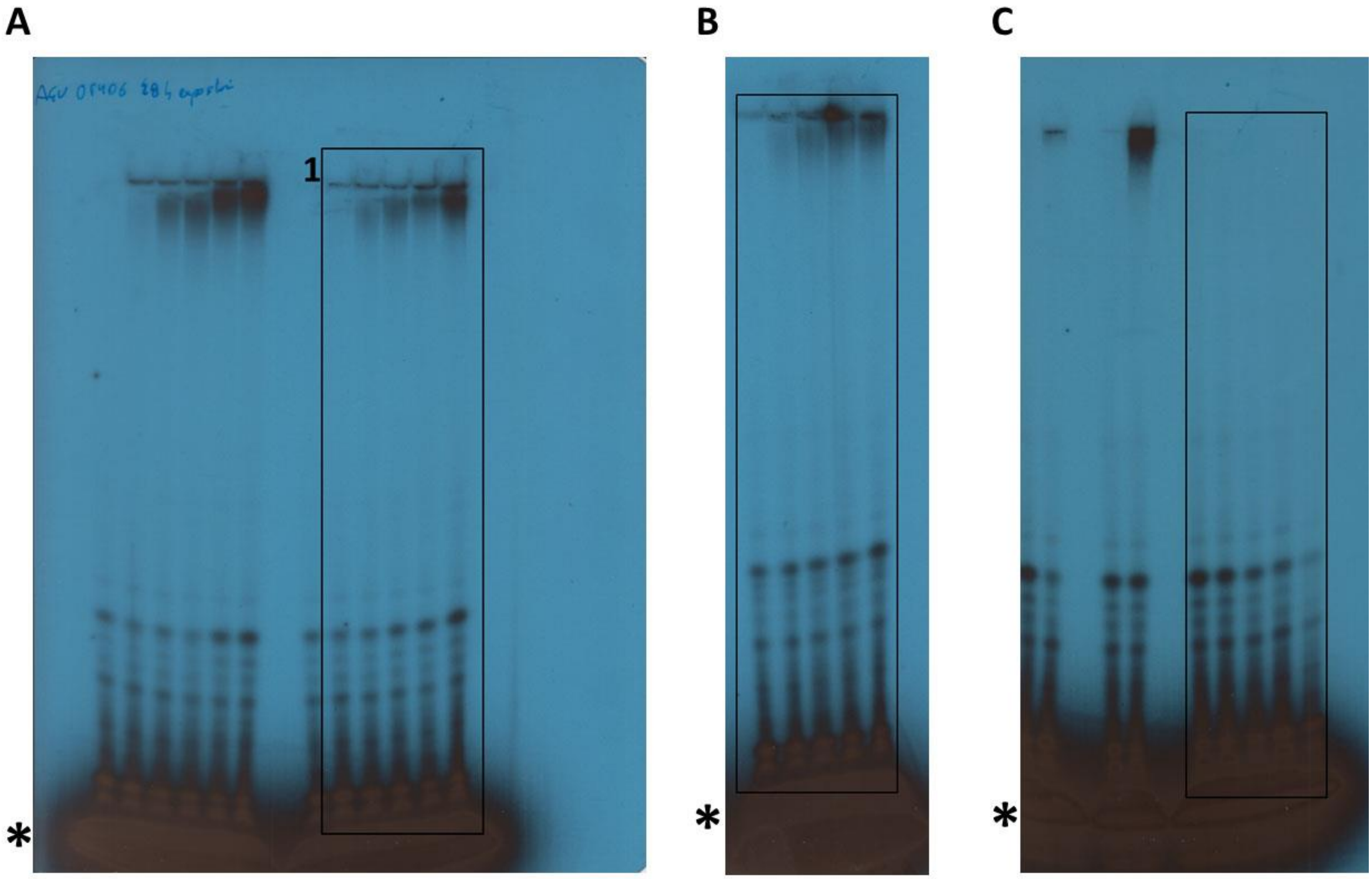

Figure S8

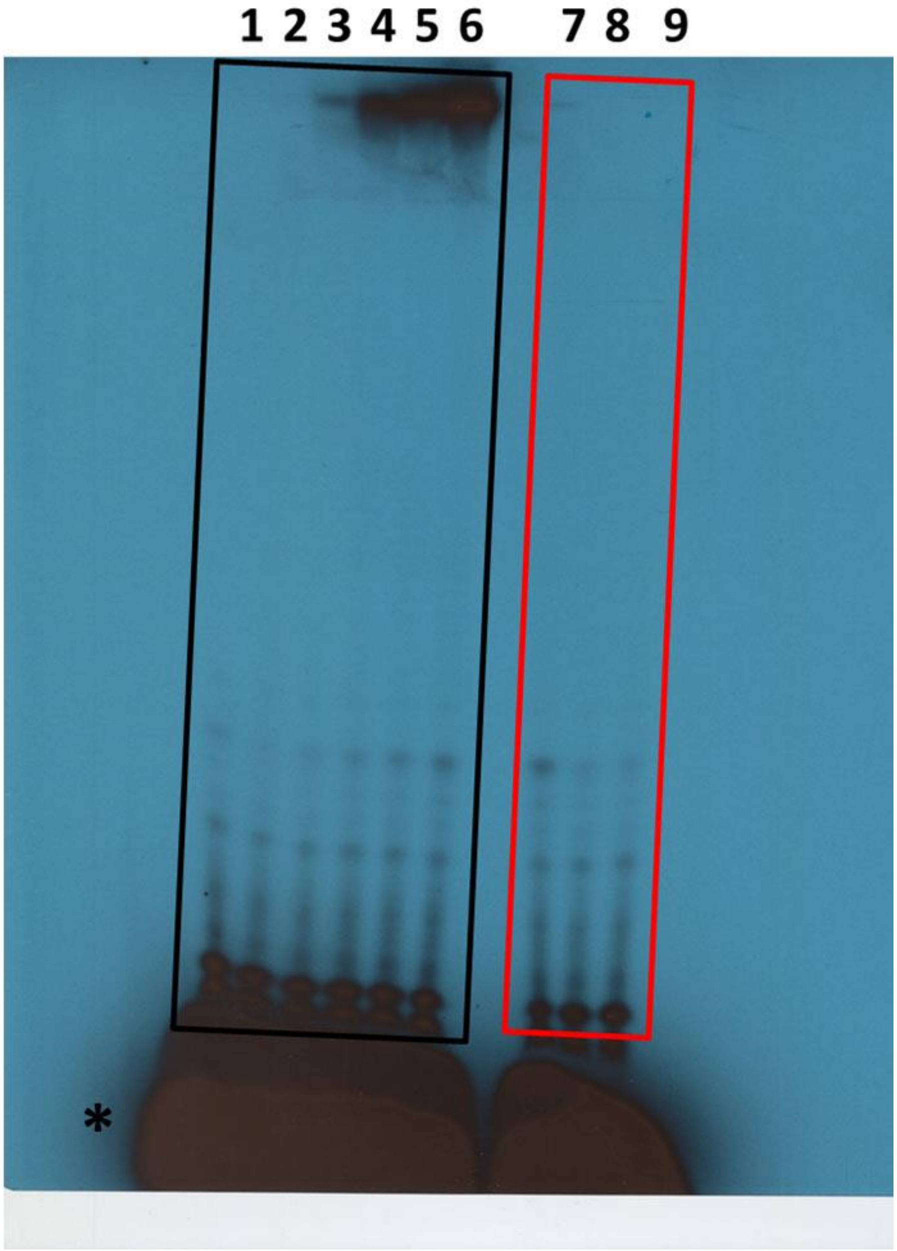

Figure S9

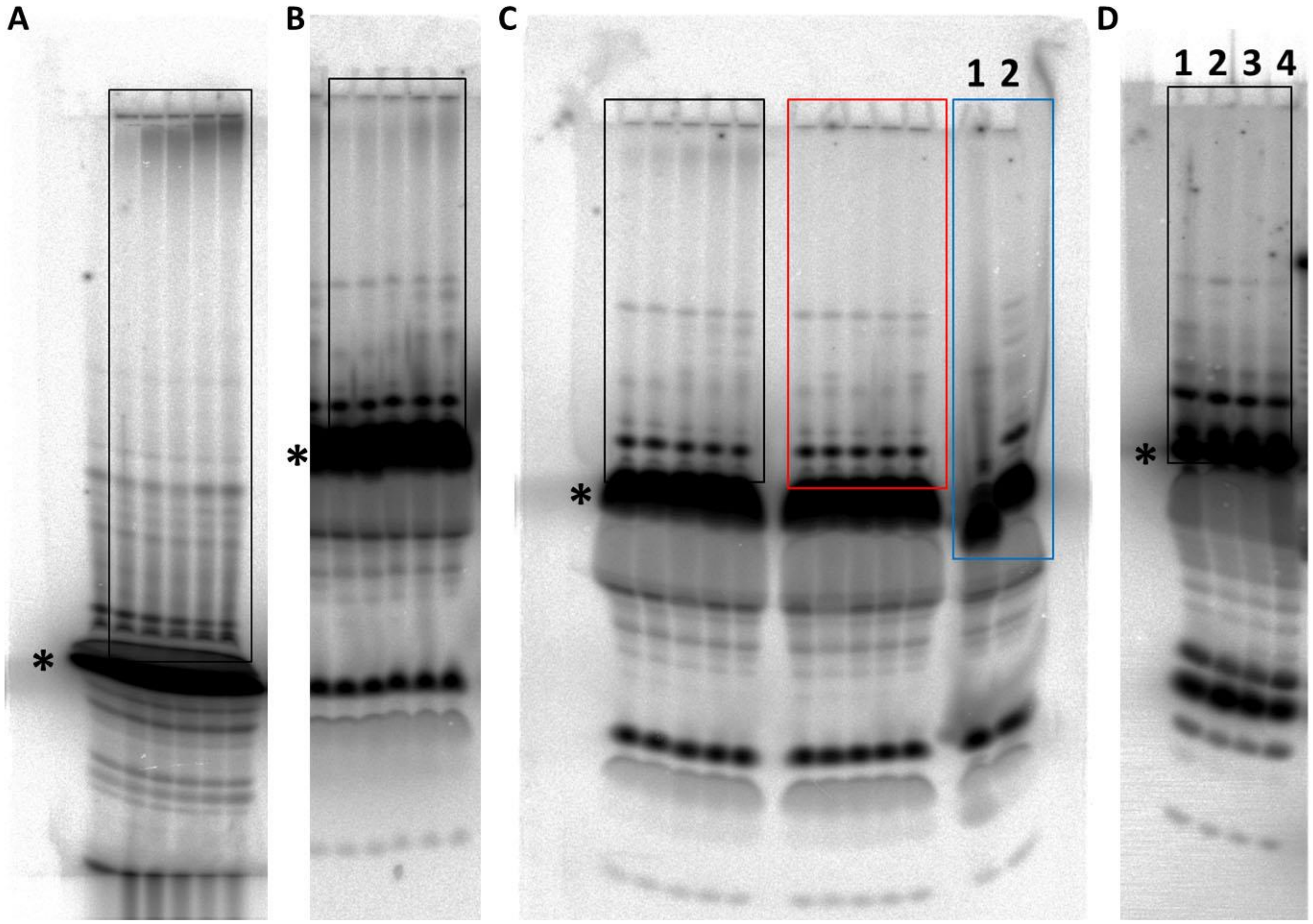

Supplement: Supplementary file 1 — Supplementary Information [file 41598_2019_41998_MOESM1_ESM.pdf]
